# Health-Promoting Properties of Anthocyanins from Cornelian Cherry (*Cornus mas* L.) Fruits

**DOI:** 10.3390/molecules29020449

**Published:** 2024-01-17

**Authors:** Iwona Szot, Grzegorz P. Łysiak, Bożena Sosnowska, Justyna Chojdak-Łukasiewicz

**Affiliations:** 1 Subdepartment of Pomology, Nursery and Enology, Institute of Horticulture Production, Faculty of Horticulture and Landscape Architecture, University of Life Sciences in Lublin, Głęboka 28, 20-612 Lublin, Poland; szoti@autograf.pl; 2 Department of Ornamental Plants, Dendrology and Pomology, Faculty of Horticulture and Landscape Architecture, Poznan University of Life Sciences, Dąbrowskiego 159, 60-594 Poznań, Poland; glysiak@up.poznan.pl; 3 Department of Biotechnology, Microbiology and Human Nutrition, Faculty of Food Science and Biotechnology, University of Life Sciences in Lublin, Skromna 8, 20-704 Lublin, Poland; 4 Department of Neurology, Wroclaw Medical University, Borowska 213, 50-566 Wrocław, Poland; justyna.chojdak-lukasiewicz@umw.edu.pl

**Keywords:** healthy properties of fruit, antidiabetic, anticancer, antioxidant, antimicrobial properties

## Abstract

The cornelian cherry is a plant that annually provides fruits, drupe-type, ranging in color from yellow through pink, red, carmine, and almost black. Cornelian cherry bears abundant fruit in temperate climate conditions, which means that its dark-colored fruits can be treated as an excellent source of anthocyanins. After consuming, anthocyanins have a protective function in the human body. Raw fruit extracts and their pure isolates, rich in anthocyanins, have a wide spectrum of health-promoting properties. This review focuses on the health-promoting properties of anthocyanins from fruits of cornelian cherry, documented in research conducted in vitro, in vivo, and in humans. The results obtained so far confirm the beneficial effects of anthocyanins on the blood parameters, whose values are important in predicting and assessing the risk and progression of cardiovascular and metabolic diseases. A beneficial effect on molecular and histopathological changes in target organs such as the heart, brain, kidneys, and liver has also been demonstrated. Anthocyanins from cornelian cherry have a strong antioxidant effect, which explains their protective effects on organs and anticancer effects. Moreover, they have antiglycemic, antihyperlipidemic, anti-inflammatory, and antimicrobial properties. The work highlights the perspectives and directions of necessary research.

## 1. Introduction

A growth in allergic reactions to synthetic chemical drugs and their undesirable side effects has increased interest in medicinal products of plant origin. Natural plant pigments—anthocyanins—are a promising group of health-promoting bio-substances. Anthocyanins, classified as polyphenols, have a structure that is characteristic of glycosides. An anthocyanin particle is normally composed of an aglycone (called anthocyanidin), from one to several sugar substituents and one or more acyl groups. Anthocyanins are so-called natural non-nutrients of plant origin, but in the human body, they have a protective role [1]. Consumption of anthocyanin-containing products triggers dynamic and complex processes such as absorption, digestion, metabolism, and excretion [2,3]. Raw extracts from fruits and other parts of the plant and their pure, anthocyanin-rich isolates show a wide spectrum of pharmacological effects [4,5].

They have anti-inflammatory and antioxidant properties, regulate apoptosis [6,7], participate in the activation of antioxidant and detoxifying enzymes [8], in the induction of signals and activation of receptors [9], and are involved in cellular interactions [10]. Through various mechanisms of action, anthocyanins have beneficial effects on many systems and organs, the improvement of which prevents or treats common diseases such as diabetes, atherosclerosis, cancer, and others [11].

Due to their common occurrence in plants, anthocyanins are present in the human diet. Quantitative and qualitative differences in the anthocyanin composition of fruit imply that these characteristics depend on plant species [12], cultivar [13], growth conditions [14], ripeness, storage [15], and processing methods [16]. The main sources of anthocyanins in a temperate climate zone are grapes, chokeberry, blackcurrant, and elderberry. Another very good source can be cornelian cherry (*Cornus mas* L.), particularly dark-colored fruits. Cornelian cherry grows in southern Europe and south-east Asia. This plant comes from the foothills of the Caucasus, and from there, it spreads over Romania, Bulgaria, Italy, Turkey, and the inland European continent [17]. On a commercial scale, it is mainly harvested from its natural habitats in Turkey, Iran, Azerbaijan, and Georgia [18]. Due to the health-promoting properties and various uses of its fruits, this plant has been raising increased interest among scientists [19]. Refining the methods of propagating and growing cornelian cherry can contribute to increasing its commercial production scale [20]. The fruits can be used in the food, pharmaceutical, and cosmetic industries, in particular due to their anthocyanin content.

The aim of the work is to analyze the current development of science regarding the health-promotion effects of anthocyanins derived from cornelian, emphasizing that the fruits of this plant, which are uncommon in the human diet, deserve to be consumed as often as possible.

## 2. Methods

**Figure 1:**
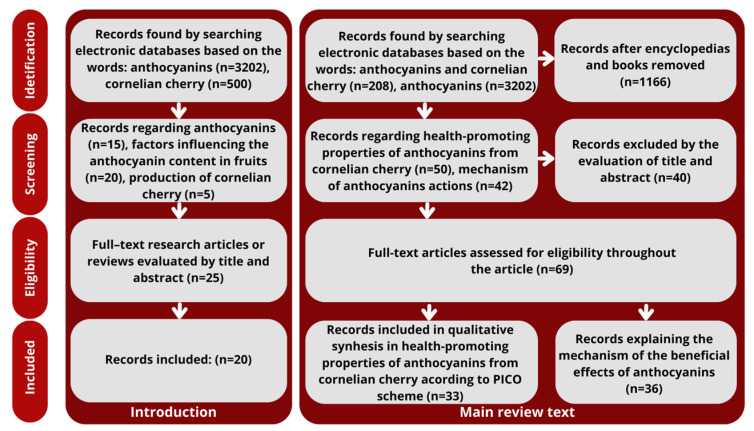
Flow chart describing the selection process of literature used in the review.

We selected reference literature using keywords such as cornelian cherry, *Cornus mas*, anthocyanins, and health-promoting properties of fruit (Figure 1) from the Web of Science, Science Direct, PubMed, and Google Scholar databases. The following tools were used: controlled vocabulary (MeSH/Emtree), logical operators, and proximity—for optimal connection sensitivity (systematicity) and precision (accuracy) of the search. We limited the search to experimental works, systematic reviews, and meta-analyses, excluding books and encyclopedias, but we did not apply any time constraints. The clinical question was formulated according to the PICO scheme, dividing the problem into four parts:

- population/patient/problem (P);
- intervention (I);
- comparison (C);
- outcome (O).

The question was: “In patients/populations (P), what is the effectiveness of drain anthocyanins (I) compared to another drug/control (C) in relieving symptoms (O)”? The dates of issue of publications covered by our review ranged from 1999 to 2023.

## 3. Bioavailability and Bioefficacy of Anthocyanins Derived from Cornelian Cherry

**Figure 2:**
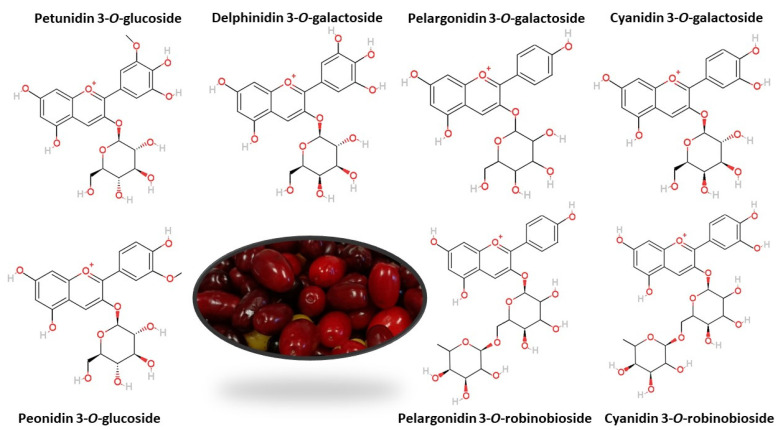
The most common anthocyanins in cornelian cherry fruits.

Anthocyanins are responsible for fruit color from pink through red to nearly black. Therefore, their level is the highest in dark-colored fruit: from 200 to 1560 mg·100 g^−1^ FW in elderberry, from 506 to 1000 mg·100 g^−1^ FW in chokeberry, from 8 to 750 mg·100 g^−1^ FW in red grapes, from 82.5 to 530 mg·100 g^−1^ FW in blueberry, and from130 to 400 mg·100 g^−1^ FW in blackcurrant [21]. Cornelian cherries contain from 5.8 to 442.11 mg·100 g^−1^ FW of anthocyanins [22,23]. Different anthocyanin content of individual fruit cultivars is also associated with their different color [24]. Yellow-colored cornelian cherries do not contain anthocyanins at all [25]. Over 650 different anthocyanins have been isolated in plants with six possible aglycon structures (anthocyanidins) most frequently occurring in natural conditions: pelargonidin, cyanidin, peonidin, delphinidin, petunidin, and malvidin [26]. Qualitative analyses of anthocyanins in cornelian cherry most often reveal the presence of cyanidin 3-*O*-galactoside [27,28,29,30], cyanidin 3-*O*-robinobioside [25,31,32], pelargonidin 3-*O*-galactoside [27,28], pelargonidin 3-*O*-robinobioside [25,31] and delphinidin 3-*O*-galactoside [29,33]. In addition, there are peonidin 3-*O*-glucoside [33,34] and petunidin 3-*O*-glucoside [31,33], with no reports of anthocyanins derived from malvidin (Figure 2).

Anthocyanins are among the few plant polyphenols that can be determined in plasma in their original, intact form as glycosides. Until recently, it was believed that anthocyanins have very poor bioavailability, and <1% of their consumed amount reaches the plasma. However, the estimation of bioavailability is limited by the lack of identification of anthocyanin metabolites and degradation products [10,35].

The biological activity of anthocyanins depends on their absorption by the human body and on metabolic processes. Anthocyanins show hydrophobic and electrostatic interactions with human albumin, preferred by hydroxyl groups in contrast to methyl groups. Studies demonstrate limited absorption of these compounds from food, as their concentration in blood plasma ranges from nM to μM. Researchers argue that anthocyanin glycosides are absorbed by a peculiar carrier—most likely a Na+ ion-dependent glucose transporter [36]. Anthocyanins and their metabolites remain in urine for up to 24 h after intake. Another factor involved in anthocyanin absorption is bilitranslocase—a plasma membrane carrier of organic anions found in the epithelial cells of the gastric mucosa [37]. Alternatively, they may also transform into glucuronide or sulfo-conjugate derivatives. Anthocyanin absorption takes place mainly in the stomach and small intestine [38]. Enzymes responsible for anthocyanin biotransformation include UDP-glucuronyl transferase, UDP–glucose dehydrogenase, and catechol-*O*-methyltransferase (COMT). They are present in the liver, small intestine, and kidneys and—depending on the chemical structure of anthocyanins—can modify them in various ways [39]. Due to this, both—primary anthocyanins and their secondary metabolites—can be detected in human urine and blood [40]. Differences in the concentrations of anthocyanins and their metabolites in the urine suggest that absorption of these pigments depends on their chemical structure, type and levels of substituted sugar radicals, and acylation method. Researchers demonstrated that binding with human albumin is more favorable for anthocyanins than it is for anthocyanidins [2]. It should be underlined that anthocyanin uptake and utilization also depend on external factors and individual traits such as age and different levels of stress [41].

David et al. [42] discovered that anthocyanins in cornelian cherries can remain in the human body for a long time. Their study, simulating a digestive process in vitro, evaluated the stability of anthocyanins derived from cornelian cherry during passage through the upper alimentary tract. Cornelian cherry extract was rich in anthocyanins such as cyanidin 3-*O*-galactoside, pelargonidin 3-*O*-glucoside, and pelargonidin 3-*O*-rutinoside. They demonstrated that gastric digestion had no significant effect on the levels of anthocyanins, and only intestinal digestion materially reduced their content and antioxidant activity. These discoveries suggest that cornelian cherries are an important source of anthocyanins in a human diet. They can have a beneficial effect on gastric health, while the products of their degradation and their metabolites can act as antioxidants in the small intestine. High levels of ingredients in fruits do not always go hand in hand with the human body’s capability of utilizing them.

## 4. Health-Promotion Effects of Anthocyanins Derived from Cornelian Cherry

The beneficial effects of anthocyanins from cornelian cherry fruit have been studied in in vitro (Table 1), in vivo (Table 2), and human experiments (Table 3).

### 4.1. Anti-Inflammatory Properties

According to the current state of knowledge, chronic inflammatory processes are involved in the pathogenesis of many diseases, including civilizational diseases. Seeram et al. [43] (Table 1) examined the anti-inflammatory properties of anthocyanins derived from cornelian cherries, which involved inhibiting cyclooxygenase. Cyclooxygenase enzyme transforms arachidonic acid derived from the structural phospholipids of the cell membrane into prostaglandins, prostacyclin, and thromboxane, responsible for inflammatory reactions. Isoforms of cyclooxygenase COX-1 and COX-2 occur in the human body. Conventional non-steroid anti-inflammatory drugs inhibit both cyclooxygenases. The cyanidine glycoside aglycone, cyanidin, acted as a strong COX-1 and COX-2 inhibitor [74,75], although anthocyanins from *C. mas* did not show as good cyclooxygenase inhibitory activity as ibuprofen and naproxen [43].

### 4.2. Antioxidant Properties

Similar to other polyphenolic compounds, anthocyanins show a strong antioxidant effect, i.e., they are capable of neutralizing free radicals and preventing oxidative stress. The antiradical activity of anthocyanins is boosted by the number of hydroxyl groups in ring B and the arylation of sugar residues by phenolic acids. In addition, anthocyanins are capable of chelating metal ions (e.g., copper) thanks to the presence of hydroxyl groups in the C ring. They also inhibit lipid peroxidation and fatty acid autooxidation. The antioxidant potential of anthocyanins is higher than, for example, that of vitamins C and E and beta-carotene. The positive effect of anthocyanins on health is mainly due to their antioxidant capacity and inhibitory effect against certain enzymes [76].

Sarma and Sharma [77] demonstrated that anthocyanins can protect the genetic material against oxidative damage through co-pigmentation with DNA. Szczepaniak et al. [78] study showed that ethanolic extracts had stronger power to interact with dsDNA. The presented study also showed that different cultivars of cornelian cherry may have various or no genoprotective effects. The differences are based on the different quantitative content of individual phenolics in the prepared extracts and different energy of interaction between them and modeled nucleotides. In the study of Milenković-Andelković et al. [29] an extract from cornelian cherry fruit is characterized by a high content of total phenolic compounds and high antioxidant activity (Table 1). The extract was rich in completely different anthocyanins, but the phenolic group was not directly related to their strong antioxidant activity, suggesting that other compounds (other phenols, ascorbic acid, etc.) present in the extracts are also involved in the antioxidant activity.

Eshaghi et al. [49], in their study on mice, demonstrated that cornelian cherry extract supplemented at 500 and 1000 mg·kg^−1^ prevented sperm damage caused by methotrexate-induced oxidative stress. This protective mechanism involves eradicating free radicals and increasing the body’s antioxidant capacity (Table 1).

Odžaković et al. [45] performed several tests, such as DPPH, ABTS, and OH radical neutralization, to determine the antioxidant capacity of several ecotypes of cornelian cherry growing in various regions of Bosnia and Herzegovina (Table 1). Ecotype CC3 from the Drinić area featured the highest level of monomeric anthocyanins (1.40 mg CyGE·g^−1^ FW) and the highest capacity of inhibiting free radicals (IC50^DPPH^ = 262.19 mg·mL^−1^; IC50^ABTS^ = 76.78 mg·mL^−1^; IC50^OH^˙ = 102.31 mg·mL^−1^). Pearson’s correlation coefficient implied a strong impact of the total content of monomeric anthocyanins and vitamin C on OH radical neutralization. They concluded that anthocyanin content, and hence fruit healthiness, may depend on the cornelian cherry growing environment, as the Drinić area is characterized by a lower mean precipitation rate, lower mean number of sunny days, and lower mean temperature than the Ljubomir and Koravlica areas where other cornelian cherry samples originated.

### 4.3. Protective Effects on Human Body Organs

#### 4.3.1. The Cardioprotective Effects

Eshaghi et al. [49], in their studies on rats, evaluated the protective effect of cornelian cherries on the heart by reducing oxidative stress triggered by a carbon tetrachloride injection (Table 2). Carbon tetrachloride induces oxidative damage to cardiac tissues, which is corroborated by increasing levels of lactate dehydrogenase and creatine kinase (enzymes responsible for ATP regeneration) and malondialdehyde (MDA, membrane lipid peroxidation marker) and decreasing antioxidant enzyme levels. Cornelian cherry extract at concentrations of 300 and 700 mg·kg^−1^ had a beneficial effect on the heart, which could be associated with its capacity to reduce cell membrane lipid peroxidation, restore enzymatic defense against oxidation, and increase the bioenergetics of cardiac tissues. Anthocyanins can inhibit fat oxidation, acting as hydrogen atom donors. This is because some O–H bonds in polyphenolic compounds are weaker than C–H bonds in fats. The hydroxyl bond dissociation energy is significantly lower than in water. This is associated with the multitude of mesomeric structures in an emerging anthocyanin radical. The ROO* radical derived from lipids can more easily detach a hydrogen atom from an anthocyanin molecule than from another fat particle. Then, the radical reaction is complete. Due to their lipid oxidation-suppressing ability, anthocyanins restore blood flow in the heart in case of inadequate blood supply.

#### 4.3.2. The Liver-Protective Effects

One of the major health issues is liver damage, which deteriorates the body’s metabolic functions. Anthocyanins have demonstrated a protective effect on liver cells (hepatocytes). Moayed Alavian et al. [50] confirmed that cornelian cherry extract has a protective effect against carbon tetrachloride-induced lipid peroxidation in the liver (Table 2). They demonstrated a considerable decline in the content of malondialdehyde (MDA) in the liver as a lipid peroxidation marker. They found that anthocyanins increase cell membrane stability and normalize biochemical profile variations. Somi et al. [51] also established that cornelian cherry extract administered to rats orally prevented liver damage induced by oxidative stress triggered by a carbon tetrachloride injection. Saei et al. [52], who induced liver damage in rats with methotrexate, also observed a hepatoprotective effect of an oral application of cornelian cherry extract. In their study, cornelian cherry extract (700 and 1400 mg·kg^−1^) significantly prevented methotrexate-induced variations in biochemical parameters such as alanine transaminase, alkaline phosphatase, and lactate dehydrogenase activity. The lipid peroxidation level in the liver decreased significantly compared to the control group.

Sangsefidi et al. [68,69] investigated the possibility of using anthocyanins derived from cornelian cherry at a dose of 20 mL·day^−1^ over 12 weeks on changes in the markers of non-alcoholic fatty liver disease (Table 3). They measured alanine aminotransferase, aspartate aminotransferase, and cytokeratin 18 levels, liver fat, and fibrosis score. They did not identify any significant differences between the examined groups, although the level of cytokeratin 18 in the anthocyanin group decreased. The fibrosis score in the placebo group significantly increased. The authors concluded that cornelian cherry extract could prove useful in the treatment of non-alcoholic fatty liver disease, but this requires continued studies in a larger population of patients and with a higher anthocyanin supply.

#### 4.3.3. The Renal Protective Effects

Es Hagi et al. [53], in their study involving rats, established that cornelian cherry extract can prevent kidney damage triggered by oxidative stress induced by carbon tetrachloride (CCl_4_) (Table 2). Some rats received *C. mas* fruit extract (CMFE) orally, at a dose of 300 and 700 mg·kg^−1^ before CCl_4_ injection, and some at the same doses 2, 6, 12, and 48 h after CCl_4_ poisoning. The study proved that CMFE alleviated the toxic effects of CCl_4_. Cornelian cherry fruit had a positive effect on carbon tetrachloride-induced changes in lipid peroxidation and antioxidant defense. It was concluded that anthocyanins from CMFE could scavenge CCl_4_ free radicals generated by the P450 enzyme system, therefore reducing oxidative damage to kidney tissues. Based on histopathological examinations, the protective effect of CMFE was demonstrated, as it prevented serious morphological abnormalities of the kidneys in the glomerular and tubular compartments.

#### 4.3.4. The Brain-Protective Effects

Poor nutrition, immobility, obesity, and aging increase the risk of neurodegenerative disorders such as Alzheimer’s Disease (AD). The aging brain finds it increasingly difficult to transmit signals between neurons, which leads to deteriorated memory, loss of concentration, and thinking problems. Anthocyanins derived from cornelian cherry improve cerebral circulation (nutrient and oxygen supply to the brain), which stops or reduces the rate of brain degeneration processes. Darbandi et al. [55] examined the effect of anthocyanins derived from *C. mas* on memory in an experimental model of Alzheimer’s Disease induced by streptozotocin injected into the cerebral ventricles of rats (Table 2). Anthocyanin treatment improves memory, depending on dosage. They emphasized that the optimum dose of the cornelian cherry extract (10 mg·kg^−1^) had a positive effect on remembering, simultaneously reducing the glucose and triglyceride levels of blood plasma.

Furthermore, anthocyanins can protect the brain during oxidative stress. Cerebral tissue is prone to oxidative stress due to the high energy requirement and high lipid and iron levels. It is also sensitive to catecholamine oxidation and reduced levels of endogenous antioxidants. The location of catalase in the main parts of the brain is uneven. Only a small part of the whole brain, which contains noradrenergic, dopaminergic, and serotonergic neurons, features particularly high catalase activity. Francik et al. [54] tested rats for the neuroprotective properties of cornelian cherry fruit under oxidative stress triggered by an unbalanced diet, with fructose or fat being its predominant components (Table 2). The addition of cornelian cherry stimulated the efficiency of paraoxonase enzyme, both in the brain tissue and in blood plasma, and increased the protection of the nervous system against a high level of protein carbonyl groups.

#### 4.3.5. Vision Protective Effects

Anthocyanins also have very good effects on vision. This effect is particularly explicit in the iris, where they reinforce blood vessels and improve ocular microcirculation, stimulate the production, and accelerate the regeneration of visual purple—rhodopsin (a very important substance in the seeing process). Therefore, an anthocyanin-rich diet improves the quality and clarity of vision and reduces the incidence rate of macular degeneration. One of the most common old-age diseases in present-day societies is glaucoma. This is strongly correlated with increased intraocular pressure (IOP), which can permanently damage vision in the affected eye [79]. 

Szumny et al. [67], on an animal model (New Zealand rabbits), compared the effect of dried cornelian cherry (*C. mas*) with a polarized polyphenolic fraction rich in iridoids and anthocyanins on decreasing IOP (Table 2). The dominant iridoid was loganic acid (50%), and the dominant anthocyanin was pelargonidin 3-galactoside (7%). Other ingredients were also an iridoid compound called cornuside and anthocyanins, such as cyanidin 3-*O*-galactoside, cyanidin 3-*O*-robinobioside, and pelargonidin 3-*O*-robinobioside. They found that a purified 0.7% loganic acid solution had a stronger hypotensive effect on intraocular pressure than the fraction rich in anthocyanins and iridoids. This effect can be compared with that of timolol, which is commonly used in ophthalmology.

### 4.4. Anticarcinogenic Activity

The protective effects of anthocyanin compounds against tumors stem from their antioxidant properties and involve neutralizing reactive oxygen species such as singlet oxygen, hydroxyl radicals, and superoxide anion radicals. The results obtained indicate that the substitution pattern at the β-ring could interfere with the anthocyanin-modulated signaling cascades involved in the regulation of colon cancer cell growth [80]. In addition, anthocyanins have a selective impact on cancerous cells, but they show little or no influence on normal cells [81]. Yousefi et al. [44] assessed the toxicity of water and alco-hol-based extract of cornelian cherry featuring high antioxidant activity in relation to human cancer cells in vitro (Table 1). They found that the extract is highly capable of inhibiting the proliferation of various cancer cells (non-small cell lung cancer, breast adenocarcinoma, ovarian cancer, and prostate cancer) irrespective of dosage, which suggests that evaluating the optimum biological dose is more significant than the using of maximum tolerated dose. The cancer cells responded better to the lower than 250 μg·ml-1 doses of cornelian cherry extract and caused >80% cell growth inhibition.

Odzankovic et al. [45] examined the antiproliferative properties of extracts from four cornelian cherry ecotypes growing in Bosnia and Herzegovina. Extracts from cornelian cherry with the highest content of monomeric anthocyanins (CC3) and the highest total polyphenol content (CC4) featured the highest capability of inhibiting cell line growth of breast adenocarcinoma, cervical squamous cell carcinoma, and lung adenocarcinoma (Table 1).

### 4.5. Antihyperglycemic Effects

Chronic hyperglycemia, accompanied by carbohydrate, lipid, and protein metabolism disorders due to the insufficient release or effect of insulin, leads to the development of a dangerous metabolic disease, i.e., diabetes mellitus. Anthocyanins affect carbohydrate metabolism through various mechanisms [82]. 

Soltani et al. [70] observed that daily consumption of *C. mas* L. fruit extract improves the control of glycemia by increasing the blood insulin level in adult type 2 diabetes patients (Table 3). Anthocyanins derived from cornelian cherry improve insulin sensitivity through complex biochemical mechanisms. Among other effects, they can inhibit the efficiency of hydrolase enzymes (glucosidases) by cutting the bonds between individual glucose particles in polysaccharides. Glucosidases include alpha-glucosidase—an enzyme that breaks down oligosaccharides and polysaccharides into monosaccharides in the brush border of the small intestine. Dzydzan et al. [25] suggest that compounds found in *C. mas* fruit may disturb the digestion or absorption of sugars in the small intestine, which will decrease blood glucose levels. Capcarova et al. [59] investigated the effect of cornelian cherry on glycemia and diabetes symptoms in obese Zucker fatty rats (Table 2). They found a significant decline in glucose levels after oral administration of *C. mas* at 1000 mg·kg^−1^ body weight.

Correct glucose concentration in case of insulin resistance increases insulin secretion by pancreatic beta-cells (hyperinsulinism). Persistent hyperinsulinism in type 2 diabetes patients exhausts the insulin production capacity of these cells. Jayapraksam et al. [46] compared the capacity of anthocyanins derived from cornelian cherry: cyaniding 3-glucoside, delphinidin 3-glucoside, cyaniding 3-galactoside, and pelargonidin 3-galactoside, with that of anthocyanidins derived from other fruit: cyanidin, delphinidin, pelargonidin, malvidin and petunidin, to stimulate insulin secretion by the pancreatic cells of rodents (INS-1 832/13) in vitro (Table 1). They analyzed the compounds at glucose concentrations of 4 and 10 mM. Delphinidin 3-glucoside and cyaniding 3-glucoside were the most effective insulin secretion stimulants among anthocyanins and anthocyanidins at tested glucose concentrations. Pelargonidin 3-galactoside and its aglycone, pelargonidin, increased insulin secretion 1.4 times at a glucose level of 4 mM. Other anthocyanins and anthocyanidins they tested showed only a marginal impact on insulin at glucose concentrations of 4 and 10 mM. This implies that the number of hydroxyl groups in ring B of anthocyanins plays an important role in terms of their capacity to stimulate insulin secretion. Another study [60] evaluated the effect of anthocyanins and ursolic acid on insulin resistance in C57BL/6 mice fed high-fat diets. Anthocyanins supplemented at 1 g·kg^−1^ high-fat diet and ursolic acid at 500 mg·kg^−1^ high-fat diet prevented glucose intolerance developed due to high-fat diet. Mice treated with anthocyanins and ursolic acid showed unusually elevated insulin levels. These findings suggest that anthocyanins and ursolic acid increase insulin sensitivity and/or insulin secretion.

Chronic hyperglycemia exposes type 2 diabetes patients to the risk of complications such as diabetic bone disease. Omelka et al. [56] explored the effect of cornelian cherry fruit pulp fed to type 2 diabetes rats on their bone quality parameters (Table 2). They confirmed the hypolipidemic effect of the fruit and observed its protective effect on bones. Cornelian cherry pulp at 1000 mg·kg^−1^ body weight had a positive effect on femoral weight, cortical bone thickness, relative volume of trabecular bone, and trabecular thickness. They argued that anthocyanins present in fruit prevented oxidative stress, which is the main reason for bone damage and deterioration of bone strength in chronic hyperglycemia associated with diabetes mellitus.

### 4.6. Hypolipidemic Effects

Lipid disorders are a group of conditions that result in abnormal blood lipid levels. They are frequently present in diabetes, obesity, and metabolic syndrome.

Asgary et al. [71] succeeded in beneficially modifying the lipid profile by supplementing anthocyanin-rich *C. mas* powder to dyslipidemic children and adolescents (Table 3). Danielewski et al. [64] confirmed that cornelian cherry extract could potentially reduce metabolic disorders associated with obesity and atherosclerosis. They fed male New Zealand rabbits with diets supplemented by 1% cholesterol over 60 days (Table 2). In addition, one group received cornelian cherry extract at 10 mg·kg^−1^ body weight, and another at 50 mg·kg^−1^ body weight. They determined the levels of cholesterol, triglycerides, adipokines, apolipoproteins, the intima and media (I/M) diameter in the thoracic and abdominal aorta, PPAR-α and PPAR-γ expression in the aorta, and LXR-α expression in the liver. They observed that the administration of cornelian cherry extract resulted in an enhancement in the expression of all tested transcription factors. Additionally, it decreased triglycerides (TG), leptin and resistin, and increased adiponectin levels. The decrease of TG under the influence of anthocyanins can be attributed to the reduction of TG-rich blood serum particles containing apo B and apo C-III. In addition, a significant reduction in the I/M ratio was observed for both the thoracic and abdominal aorta. Adipose tissue releases several mediators called adipokines (e.g., adiponectin, leptin, and resistin). Adipokines control various metabolic processes and can participate in the development of metabolic diseases such as type 2 diabetes and cardiovascular diseases. Obese people showing high leptin and resistin levels are exposed to the risk of developing insulin resistance, and adiponectin could prevent this. Canadian and Chinese studies corroborated the anthocyanin effect in reducing leptin concentrations [83]. 

Sozański et al. [61], in a study on rabbits with diet-induced hyperlipidemia (fed 1% cholesterol), compared the effect of cornelian cherry lyophilizate (100 mg·kg^−1^ body weight) and symvastatin (5 mg·kg^−1^ body weight) in preventing atherosclerosis (Table 2). They identified five anthocyanins and two iridoids in fruits, to which they attributed a beneficial effect on decreasing triglyceride levels of blood serum and preventing the development of atherosclerotic lesions in the thoracic aorta. Only a diet enriched with cornelian cherry fruits contributed to increasing PPAR-γ expression in the liver, which implies that its hypolipidemic effect can be due to enhanced catabolism of fat compounds. Cornelian cherry showed a significant protective effect on diet-induced oxidative stress in the liver and standardized elevated pro-inflammatory cytokine levels of blood serum.

Another experiment by Sozański et al. [63] compared the effect of anthocyanins and iridoids derived from cornelian cherries on diet-induced atherosclerosis. Anthocyanins decreased total cholesterol, LDL, and triglyceride levels and increased HDL levels (Table 2). Loganic acid induced significant changes in triglyceride and HDL levels only. Anthocyanins improved the thoracic aorta diameter to a greater extent than iridoids by reducing the intima thickness and the I/M ratio. Anthocyanins also enhanced PPAR-γ expression in the liver more strongly, but their anti-inflammatory effect was weaker. Thus, in the production of phytopharmaceuticals, both substances should be combined to boost their efficiency. In another experiment, Sozański et al. [62] demonstrated that loganic acid and anthocyanins isolated from cornelian cherry fruits reduced the development of thoracic atherosclerosis and, to a lesser extent, abdominal atherosclerosis. Both substances decreased lipid peroxidation and oxidative stress in the liver (Table 2).

An Iranian study by Rafieian-Kopaei et al. [84] assessed the effect of cornelian cherry fruits on atherosclerosis and its risk factors in hypercholesterolemic rabbits. Powdered *C. mas* fruits added to their diets considerably increased antioxidant capacity and decreased malondialdehyde (MDA), fibrinogen, and AIP (AIP = log TG/HDL) levels of blood plasma. They also decreased TC, LDL, and TG levels and reduced atherosclerotic lesions in the aorta. Świerszczewska et al. [47] investigated the possibilities of using anthocyanins derived from cornelian cherry fruits to reduce the development of diseases accompanying hyperlipidemia (Table 2). The most effective fraction limiting the efficiency of digestive hormones (pancreatic lipase) was that containing pelargonidin 3-*O*-galactoside. Inhibiting digestive enzymes involved in breaking down fat and starch is a mechanism used in the treatment of obesity and related diseases through decreasing fat absorption and the post-meal increase of blood glucose. The study by Jayaprakasam et al. [60] evaluated the effect of anthocyanins and ursolic acid on mitigating obesity in C57BL/6 mice fed high-fat diets (Table 2). Anthocyanins in the diet contributed to 24% weight loss, irrespective of their dosage. The mice also showed decreased accumulation of lipids in the liver, including a significant decline in triacylglycerol concentration in the liver. The researchers found that anthocyanins and ursolic acid could control lipid metabolism through their effect on lipid oxidation in the liver and lipogenesis. An Iranian study [58] on diabetic rats observed considerably elevated levels of blood glucose, LDL-C, TG, AST, ALP, and ALT and decreased HDL-C compared to the non-diabetic group (Table 2). The use of glibenclamide or cornelian cherry fruit powder countervailed the above irregularities. The effect of *C. mas* fruits was comparable with that of glibenclamide at doses tested in that study. The concentrations of glucose, TG, ALP, and HDL in blood serum in the normal group changed significantly in comparison with the control group of diabetic patients. Histopathological examination revealed mild hepatitis portals in groups fed *C. mas* fruits compared with other study groups.

Iranian studies noted a unique anticoagulant effect of dried cornelian cherries. Hypercholesterolemic rabbits fed powdered cornelian cherries over 60 days showed decreased fibrinogen levels of blood plasma (Table 2). The outcomes imply that the consumption of dried cornelian cherry can be beneficial for atherosclerotic patients due to its fibrinogen-reducing effect [65].

Abdollahi et al. [66] found a significant reduction of platelet distribution width (PDW) in male rats receiving a hydromethanolic extract of cornelian cherry at 50, 200, and 400 mg·kg^−1^ body weight over three weeks (Table 2). Changes in these platelet parameters suggest inhibiting platelet activity without bone marrow suppression in the treatment groups compared with control groups. In addition, reduced PDW pointed to homogeneous size and inactivation of platelets, which testifies to the protective effects of cornelian cherry fruits on the cardiovascular system. Antioxidants decrease platelet activity by stimulating prostacyclin synthesis and eradicating superoxides that have an inhibitory effect on synthesis. Yarkosseini et al. [72] examined the effect of anthocyanins derived from cornelian cherry fruits, administered over 12 weeks at 20 mL·day^−1^, on blood pressure, anthropometric ratios, and body composition analysis of obese people suffering from non-alcoholic fatty liver disease (Table 3). Anthocyanins derived from cornelian cherry significantly decreased blood pressure and reduced adipose tissue but did not change the anthropometric ratios and body composition. The researchers found it necessary to continue research with a higher anthocyanin dosage.

Liu et al. [85] argued that anthocyanin supplementation was associated with a decrease in TC, TG, and LDL, but the levels of HDL increased compared with the control group. Anthocyanins can reduce TC in blood plasma, probably due to increased excretion of fecal neutral and acid sterols. Furthermore, anthocyanins decrease HMG-CoA hepatic reductase gene expression, which results in inhibiting cholesterol synthesis. The reduction of LDL cholesterol concentration under the influence of anthocyanins can be partly due to inhibiting cholesteryl ester transfer protein (CETP), which intermediates in depleting cholesterol esters from HDL in exchange for TG molecule mainly derived from LDL, VLDL, or chylomicrons.

Hyperglycemia and hyperlipidemia can intensify oxidative stress, change gene expression, and give rise to inflammatory processes, which leads to vascular complications. Anthocyanins have a positive effect on blood vessels (make them stronger and more flexible and decrease their permeability) and on the correct function of the vascular endothelium.

### 4.7. Antimicrobial Effects

Due to their antimicrobial properties, cornelian cherry fruits can be helpful in urinary tract infections (UTI). Dadkhah et al. [73], in Iran, examined the effect of taking pills containing 500 mg of powdered cornelian cherry fruits to prevent recurring cystitis in women aged 15 to 45 (Table 3). The first stage of the pathogenesis of this disease is uropathogenic adhesion to the urinary system cells. The anti-adhesive properties of anthocyanins derived from cornelian cherry fruits prevent cystitis by changing the adhesion of *Escherichia coli* to the epithelial cells of the urinary tract or by decreasing the adhesion of fecal bacteria.

The antimicrobial properties of cornelian cherry are not limited to fruits only. Krzyściak et al. [86] evaluated the antimicrobial potential of extracts of fruits but also of seeds, leaves, and bark. It was emphasized that the alcoholic extract from the seeds and leaves of *C. mas* had higher antimicrobial activity against *S. aureus*, *E. coli*, *P. aeruginosa*, and *C. albicans* than from the bark and fruits of this plant, indicating that not only anthocyanins influence these properties.

## 5. Safety of Anthocyanins

Certain fruits, such as elderberry, viburnum, goji berry, and rowanberry, should be consumed when they achieve proper ripeness or in a processed form because they may contain toxic alkaloids. The fruits of *C. mas* and its close relative *C. officinalis* have been consumed for thousands of years with no toxic effect ever recorded on the human body. West et al. [87] performed a genetic toxicity test and acute oral toxicity test on rats and did not identify any negative effects of cornelian cherry juice or purée.

In the opinion of EFSA (European Food Safety Authority), anthocyanins, at concentrations present in plant products, do not have any side effects on the human body. However, it should be noted that anthocyanins are also used as food additives (E-163) or consumed at increased doses as dietary supplements. Nevertheless, at the moment, we lack toxicological data that would facilitate determining the acceptable daily intake (ADI). The opinion of EFSA issued in 2013 maintains the position of the Scientific Committee on Food (SCF), stating that the use of anthocyanins as food additives is safe, provided that levels possibly supplied with diet are not exceeded. The estimated exposure to anthocyanins in the daily diet is, on average, about 0.1 mg·kg^−1^ body weight per day for adults and 0.3 mg·kg^−1^ body weight per day^−1^ for children [88]. Soltani et al. [70] did not identify any toxic effect of anthocyanins derived from cornelian cherry extract consumed over six weeks at 600 mg (Table 3).

Although anthocyanins showed multiple healthy properties, their potential adverse effect should also be taken into consideration. This is mainly associated with possible interactions with medicines, which can either increase or decrease bioavailability and, hence, the efficiency of a specific pharmaceutical. Tests involving laboratory animals and in vitro tests paid particular attention to interactions between anthocyanins and nifedipine, i.e., a medicine to treat hypertension, and diclofenac, i.e., a non-steroidal anti-inflammatory drug. Anthocyanins derived from cranberry juice inhibited drug metabolism, hence extending and intensifying its effect [89]. Such interactions have not yet been corroborated by testing on humans, or the results of experiments are ambiguous. However, when taking the above medicines, one should consider their potential interactions with anthocyanins and should not exaggerate consuming anthocyanin-rich products.

## 6. Conclusions

Anthocyanins derived from cornelian cherry have the potential to have a beneficial effect on human health. Studies have shown that anthocyanins improve blood parameters, playing an important role in medical prevention and enabling the prediction and assessment of the risk and progression of cardiovascular and metabolic diseases. In addition, they had a beneficial effect on molecular and histopathological changes in the target organs, such as the heart, brain, kidneys, and liver. Most studies determining the antioxidant, antidiabetic, hypolipemic, and protective effect of anthocyanins derived from cornelian cherry fruits on particular body organs, systems, and blood vessels involved in vivo testing of biological models such as rats, rabbits, and mice suffering from diseases induced by pharmacology or diet. In vitro tests explain the antihyperglycemic and antihyperlipidemic mechanisms behind the effect of anthocyanins derived from cornelian cherry fruits. In addition, they point to their anti-inflammatory, anticancer, and antimicrobial effects. Anthocyanins enhance insulin secretion by β-cells of pancreatic islets, increase the insulin sensitivity of tissues, slow down carbohydrate uptake in the alimentary tract, and enhance glycogen production. A diet rich in anthocyanins derived from cornelian cherry positively affects the lipid profile of blood plasma. The beneficial effect of anthocyanins derived from cornelian cherry on the lipid profile and on atherosclerotic ratios and lesions stems from their antioxidant activity, i.e., preventing lipid peroxidation by affecting suboxides, hydrogen radicals, and by chelating metal anions. Antimicrobial properties of anthocyanins can be useful in the treatment of patients resistant to antibiotics. Anthocyanins at concentrations present in plant products do not have any side effects on the body.

The various uses of cornelian cherry in fruit desserts, juices, purées, pastes, or as dried fruit make it possible to beneficially increase the biodiversity of the human diet. More studies involving human subjects are necessary to determine the optimum method of anthocyanin intake with cornelian cherry products to effectively prevent and treat specific diseases.

## Figures and Tables

**Table 1:** Health-promoting properties of cornelian cherries anthocyanins proven in in vitro tests presented according to the PICO scheme.

Health-Promoting Properties	Literature	Cells Used in the Study (Population)	Study Treatment (Intervention)	Control Treatment (Comparison)	Main Findings (Outcome)
Anti-inflammatory activity	[43]	Ram seminal vesicles	Anthocyanins of *C. mas*—juice: delphinidin 3-*O*-‚-galactopyranoside 280 ppm, cyanidin 3-*O*-‚galactopyranoside 1079 ppm, and pelargonidin 3-*O*-‚-galactopyranoside 710 ppm	Ibuprofen, naproxen,	Ibuprofen and naproxen showed 47.5 and 54.3% of COX-I and 39.8 and 41.3% of COX-II inhibitory activities, respectively, at 10 μM concentrations. Anthocyanins 1, 2, and 3 displayed 9.2, 7.6, and 5.3% COX-I and 11.7, 12.4, and 7.8% COX-II activities, respectively.
Anticancer activity	[44]	Lung non-small cell cancer; breast adenocarcinoma cell; ovarian cancer cell; prostate adenocarcinoma cell	Hydro-alcoholic extract of *C. mas:* 0, 5, 20, 100, 250, 500, 1000 μg/mL	Negative control (cells in RPMI-1640 medium)	The mean growth inhibition was 81.8%, 81.9%, 81.6%, and 79.3% in ovarian cancer, breast adenocarcinoma, prostate adenocarcinoma, and lung non-small cell cancer, respectively.
	[45]	Breast adenocarcinoma, cervix epithelioid carcinoma, lung adenocarcinoma	containing different amounts of anthocyanins depending on growth locations CC1 0.89, CC2 0.80, CC3 1.40, CC4 1.08 mg CyGE·g^−1^ FW	Negative control (Cells in DMEM medium) (PAA Laboratories GmbH, Pashing, Austria)	The antiproliferative activity of cornelian cherry fruit extracts depended on growth locations. Wild cornelian cherry (CC3) from Drinić had the highest monomeric anthocyanin content and the highest inhibition of free radicals (IC50^DPPH^ = 262.19 mg/mL; IC50^ABTS^ = 76.78 mg/mL; IC50^OH˙^ = 102.31 mg/mL) and inhibition of breast adenocarcinoma cell line growth (IC50^MCF^-7 = 1.37 mg/mL).
Antihyperglycemic effects	[46]	Rodent pancreatic β-cells (INS-1 832/13)	Purified delphinidin-3-glucoside from *C. officinalis* fruits. cyanidin-3-galactoside and pelargonidin-3-galactoside from *C. mas* fruits: 5, 10, 50, 100 and 250 μg·mL^−1^.	Negative control (cells in RPMI-1640 medium)	Delphinidin 3-*O*-glucoside, cyanidin 3-*O*-galactoside, and pelargonidin 3-*O*-galactoside were distinguished as the most effective anthocyanins to stimulate insulin secretion.
Antihyperlipidemic effects	[47]	Porcine pancreas powder	Water and ethanolic extract of *C. mas* and *C. alba*; pelargonidin 3-*O*-galactoside: 7.5 μg·mL^−1^	Negative control (Porcine pancreas powder in Tris-HCl buffer, pH 8.0)	The most active anthocyanin in the inhibition of pancreatic lipase activity was pelargonidin 3-*O*-galactoside.

CC1—cornelian cherry from Ljubomir, CC2—cornelian cherry from Koravlica, CC3—cornelian cherry from Drinić, CC4 cornelian cherry from Drvar, FW—fresh weight.

**Table 2:** Health-promoting properties of cornelian cherries and anthocyanins proven in vivo presented according to the PICO scheme.

Effects	Literature	Animals Subjected to Tests (Population)	Study Treatment (Intervention)	Control Treatment (Comparator)	Main Findings (Outcome)
Antioxidant activity	[48]	8–12 weeks male NMRI mice treated with Methotrexate (MTX)	CMFE (250, 500, 1000 mg·kg^−1^) and Vitamin E (100 IU kg^−1^)	physiologic saline	Both Vit E and CMFE were able to protect from MTX-induced effects on sperm maturity and DNA damage.
Protective effect on the heart	[49]	Rats treated with carbon tetrachloride (CCl_4_)	Pre and post-treatment CMFE 300 and 700 mg·kg^−1^	Control group without CMFE	CMFE significantly decreased the increased levels of serum lactate dehydrogenase, serum creatine kinase, and myocardial lipid peroxides and significantly increased the myocardial endogenous antioxidants (glutathione peroxidase, superoxide dismutase, and catalase) levels.
Protective effect on the liver	[50]	Wistar strain male albino rats treated with carbon tetrachloride	CMFE 200 and 500 mg·kg^−1^ and Silymarin 100 mg·kg^−1^	irrigated with drinking water	Oral administration of CMFE provided significant liver protection by reducing elevated serum enzyme levels, total serum protein, albumin, and hepatic lipid peroxidation content.
	[51]	Wistar strain male albino rats treated with carbon tetrachloride	CMFE 200 and 500 mg·kg^−1^ per 16 days and CMFE 200 and 500 mg·kg^−1^ administered 2, 6, 12, 24 and 48 h after CCl_4_ toxication	irrigated with drinking water	The activities of antioxidant enzymes (MDA, CAT, SOD, GPx) in the CCl_4_-treated group were lower than those in the normal control. The activity of these enzymes in the CMFE-treated groups increased significantly compared to the toxic group.
	[52]	Wistar strain male albino rats treated with methotrexate (MTX)	CMFE 700 mg·kg^−1^ per 7 days and Mice first day treated with MTX and then treated with 300, 700, and 1400 mg for 7 days	physiologic saline	Rats treated with MTX were characterized by significantly higher total bilirubin values and higher AST, ALT, and ALB values compared to rats treated with CMFE. The most beneficial effect on the normalization of the above-mentioned parameters after MTX administration was CMFE treatment at a dose of 1400 mg·kg^−1.^
Protective effect on the kidneys	[53]	Wistar strain male albino rats treated with carbon tetrachloride-	Prophylactic groups: CMFE 300 and 700 mg·kg^−1^, for 16 days, respectively and on the 16th day received CCl_4_ and curative groups: distilled water orally for 16 days and on the 16th day they received CCl_4_ (1 mL·kg^−1^ b.w.; 80% in olive oil), followed by CMFE 300 mg·kg^−1^ and 700 mg·kg^−1^, respectively, at 2, 6, 12, 24 and 48 h after CCl_4_ intoxication	“Sham” control for both preventive and therapeutic studies, receiving raw water and free access to food for 16 days and control for both preventive and therapeutic studies, receiving distilled water orally for 16 days, and on day 16 received olive oil daily (1 mL kg^−1^ b.w.)	Different doses of fruit extract (300 and 700 mg/kg^−1^) significantly ameliorated the alterations induced by CCl_4_ in lipid peroxidation, antioxidant defenses, and biochemical and renal lesions. The level of antioxidant enzymes such as SOD, CAT, and GPx decreased in the CCl_4_-treated group and improved by treatment with CMFE.
Protective effect on the brain	[54]	12-week Wistar strain male albino rats	Rats with fructose diet (with CMFE)	Rats with a diet enriched in fat	Addition of CMFE stimulates PON activity, both in brain tissue and in plasma, and increases the protection of the nervous system from oxidative stress by increasing the activity of CAT. Protects proteins against peroxidation, as can be shown by the level of PCG.
	[55]	Wistar rats with streptozotocin-induced Alzheimer’s Disease	Flavonoid from CMFE at 5, 10 and 20 mg·kg^−1^	saline-saline control, streptozotocine-saline control	CMFE treatment increased memory retention in a dose-dependent manner. The dose of 10 mg kg^–1^ decreased rat weight significantly.
Protective effect on bones	[56]	Zucker diabetic fatty (ZDF) rats	diabetic obese rats receiving 500 and 1000 mg·kg^−1^ b.w. of CMFE	non-diabetic lean rats	A higher dose of CMFE had a beneficial impact on femoral weight, cortical bone thickness, relative volume of trabecular bone, and trabecular thickness.
	[57]	12-week-old female Wistar Rats with ovariectomy-induced metabolic changes	ovariectomized animals receiving 17β-oestradiol; group with CMFE (50 mg·kg^−1^)	“Sham” operated group and ovariectomized control group	CMFE ameliorated ovariectomy-induced decrease in femoral and tibial bone mineral density (BMD), prevented the deterioration in Young’s modulus and flexural strength and counteracted ovariectomy-induced decrease in serum calcium level.
Antihyperglycemic	[58]	Adult male rats with alloxan-induced diabetes	glibenclamide-treated (0.6 mg·kg^−1^·day^−1^; 4 weeks) and CMFE-treated (2 g^−1^ day; 4 weeks) group	non-diabetic control and diabetic control	Diabetic rats had significantly elevated levels of serum glucose, LDL-C, TG, AST, ALP, and ALT and decreased levels of HDL-C compared to the non-diabetic group. The effects of CMFE were comparable to those of glibenclamide at the doses tested in this study.
	[59]	Zucker diabetic fatty (ZDF) rats	CMFE in two doses (500 and 1000 mg·kg^−1^ b.w., 10 weeks)	non-diabetic lean controls received only distilled water	significant decrease of glucose level after oral administration of CMFE in a dose of 1000 mg/kg bw in the pre-diabetic state of animals (until the 7th week of the experiment) and significant restriction of water intake in both CMFE groups against the diabetic control.
	[25]	Male Wistar rats with streptozotocin-induced diabetes mellitus	CMFE extracts (20 mg kg^−1^ of b.w., 14 days)	control group (healthy)	CMFE lowered blood glucose and improved glucose tolerance. Significantly decreased the amount of glycated hemoglobin (by 25%) and increased erythrocyte resistance to acid hemolysis.
Antihyperlipidemic effects	[60]	C57BL/6 mice fed a high-fat diet	mice were fed with a high-fat diet plus anthocyanins (1 g·kg^−1^) or ursolic acid 500 mg·kg^−1^)	mice were fed a normal diet	The anthocyanin showed a 24% decrease in weight gain and decreased lipid accumulation in the liver, including a significant decrease in liver triacylglycerol concentration. Anthocyanin and ursolic acid have extremely elevated insulin levels.
	[58]	Adult male rats with alloxan-induced diabetes mellitus	glibenclamide-treated (0.6 mg/kg/day; 4 weeks) and CMFE (2 g·day^−1^; 4 weeks) group	non-diabetic control and diabetic control,	Treatment with glibenclamide or CM counterbalanced significantly increased serum glucose, LDL-C, TG, AST, ALP, and ALT levels and decreased HDL-C levels in diabetic rats.
	[61,62,63]	Adult male New Zealand rabbits with high cholesterol (1%) diet-induced hyperlipidemia	CMFE (100 mg·kg^−1^ b.w., 60 days) or simvastatin (5 mg·kg^−1^ b.w., 60 days) or loganic acid (20 mg·kg^−1^ b.w.)	control group with a standard diet and group with hyperlipidemia	CMFE led to a 44% significant decrease in serum TG levels and prevented the development of atheromatous changes in the thoracic aorta. CMFE significantly increased PPARα, had a significant protective effect on diet-induced oxidative stress in the liver, as well as restored upregulated pro-inflammatory cytokines serum levels.
	[64]	Adult male New Zealand rabbits with high cholesterol (1%) diet-induced hyperlipidemia	CMFE (10 or 50 mg·kg^−1^ b.w.) or simvastatin (5 mg·kg^−1^ b.w.)	control group with a standard diet for rabbits and a group with hyperlipidemia	CMFE enhancement in PPAR-α and PPAR-γ expression in the aorta, LXR-α expression in the liver, a decrease in TG, leptin, and resistin, and an increase in adiponectin levels.
	[65]	Adult male New Zealand rabbits with high cholesterol (1%) diet-induced hyperlipidemia	Group with a standard diet containing *C. mas* powder (1 g·kg^−1^ b.w.) diet, and with a high cholesterol (1%) containing *C. mas* powder (1 g·kg^−1^) diet, and a high cholesterol containing lovastatin (10 mg·kg^−1^ b.w.) diet	control group with a standard diet for rabbits and with a high cholesterol (1%) diet	*C. mas* powder and lovastatin significantly decreased fibrinogen levels in comparison with the high cholesterol group. Furthermore, *C mas*. powder could reduce the fibrinogen level more than lovastatin
	[66]	Healthy male Wistar rats	CMFE (50, 200 and 400 mg·kg^−1^ b.w., 3 weeks)	Normal control with normal diet without any injection and placebo control and intraperitoneally received a normal saline	All doses of CMFE significantly decreased HDW and PDW vs. the control group. Only high doses caused a significant elevation in MCHC, MPV, and PCT and a significant decrease in RDW vs. the control group.
Protective effect on the eyes	[67]	Sexually mature, New Zealand white rabbits, aged between 6 and 12 months, were used: 7 males and 7 females	Intraconjunctival administration of one drop loganic acid or polyphenolic fraction of *C. mas*, corresponding to a volume of 50 µL, to the right eye	One drop of artificial tears containing 0.15% sodium hyaluronate (50 µL) was administered to the left eye as a placebo	Loganic acid (50%) and pelargonidin-3-galactoside (7%) were found as the main components of *C. mas* fraction. Therefore, the hypotensive effect was attributed to loganic acid.

ALB—albumin, ALP—alkaline phosphatase, ALT—alanine amino transferase, AST—aspartate amino transferase, CAT—catalase, CMFE—*Cornus mas* fruit extract, GPx—glutathione peroxidase, HDL-C—high, density lipoprotein cholesterol, HDW—hemoglobin distribution width, LDL-C—low-density lipoprotein cholesterol, MCHC—mean corpuscular hemoglobin concentration, MDA—malondialdehyde, MPV—mean platelet volume, PCG—protein carbaryl groups, PCT—total platelet mass, PDW—platelet distribution width, PON—paraoxonase enzyme activity, RDW—red cell distribution width, SOD—superoxide dismutase, TG—triglycerides.

**Table 3:** Health-promoting properties of cornelian cherries anthocyanins proven in human studies, presented according to the PICO scheme.

Effects	Literature	Type of Patients (Population)	Dose of Cornelian Cherry Extract and Period of Its Intake (Intervention)	Control Treatment (Comparator)	Main Findings (Outcome)
Protective effect on the liver	[68]	Patients with non-alcoholic fatty liver disease (NAFLD)	CMFE (320 mg·d^−1^ anthocyanins; 12 weeks).	Control group received the placebo, matched with the extract in terms of appearance, taste, color, and texture (but without any anthocyanins) for 12 weeks	Results indicated that anthocyanins had some impacts on NAFLD.
	[69]	Patients with non-alcoholic fatty liver disease (NAFLD)	CMFE (320 mg·d^−1^ anthocyanins; 12 weeks).	Control group received the placebo, matched with the extract in terms of appearance, taste, color, and texture (but without any anthocyanins) for 12 weeks	No significant impact of CMFE on serum ALT and AST levels, as well as hepatic steatosis among NAFLD patients. A significant reduction was observed in the levels of CK-18 among the CMFE group at the end of the study. No significant difference was found between the CMFE and placebo groups with regard to this marker. Fibrosis score increased significantly in the placebo group at the end of the study.
Antihyperglycemic	[70]	Patients with type 2 diabetes mellitus	300 mg d^−1^ anthocyanins; 6 weeks	Placebo capsules; 6 weeks	Significant increase in insulin levels and a decrease in HgbA1 C and TG levels was observed in the drug group compared to the placebo.
Antihyperlipidemic effects	[71]	Dyslipidemic children and adolescents	50 g of CMFE twice a day after lunch and dinner, 6 weeks	Diet without CMFE, 6 weeks	After week 6 of the trial, the TC, TG, LDL-C, apo B, ICAM-1, and VCAM-1 levels in the CMFE group were significantly lower, and the HDL-C and apo A-I levels were higher than at baseline.
	[72]	Patients with non-alcoholic fatty liver disease (NAFLD)	20 cc/d CMFE, 12 weeks	Placebo, 12 weeks	Treatment group, compared to the control group, showed a significant reduction in DBP and SBP. No difference between groups in weight, WC, HC, WHR, BFM, BFP, and FFM. Significant reduction in the treatment group compared to the control group in BFM and BFP.
Protective effect on the urinary system	[73]	Women with chronic cystitis (UTIs)	*C. mas* tablet 500 mg·day^−1^, 6 month	Placebo, 12 weeks	*C. mas* decreases dysuria among patients with UTIs.

AST—aspartate amino transferase, ALT—alanine amino transferase, BFM—body fat mass, BFP—body fat percent, DBP—diastolic blood pressure, FFM—fat-free mass HC—hip circumference, HDL-C—high, density lipoprotein cholesterol, ICAM-1—intracellular adhesion molecule-1, LDL-C—low-density lipoprotein cholesterol, NAFLD—non-alcoholic fatty liver disease, SBP—systolic blood pressure, VCAM-1—vascular cell adhesion molecule-1; WC—waist circumference, WHR—waist-to-hip ratio.

## Data Availability

Not applicable.

## References

[1] Cheng Y., Liu J., Li L., Ren J., Lu J., Luo F. (2023). Advances in Embedding Techniques of Anthocyanins: Improving Stability, Bioactivity and Bioavailability. Food Chem. X.

[2] Kalt W. (2019). Anthocyanins and Their C6-C3-C6 Metabolites in Humans and Animals. Molecules.

[3] Milbury P.E., Vita J.A., Blumberg J.B. (2010). Anthocyanins Are Bioavailable in Humans Following an Acute Dose of Cranberry Juice. J. Nutr..

[4] De Biaggi M., Donno D., Mellano M.G., Riondato I., Rakatoniaina E.N., Beccaro G.L. (2018). *Cornus mas* (L.) Fruit as a Potential Source of Natural Health-Promoting Compounds: Physico-Chemical Characterisation of Bioactive Components. Plant Foods Hum. Nutr..

[5] Hosseinpour-Jaghdani F., Shomali T., Gholipour-Shahraki S., Rahimi-Madiseh M., Rafieian-Kopaei M. (2017). *Cornus mas*: A Review on Traditional Uses and Pharmacological Properties. J. Complement. Integr. Med..

[6] Jing N., Song J., Liu Z., Wang L., Jiang G. (2020). Glycosylation of Anthocyanins Enhances the Apoptosis of Colon Cancer Cells by Handicapping Energy Metabolism. BMC Complement. Med. Ther..

[7] Tenuta M.C., Loizzo M.R., Tundis R., Dugay A., Bouzidi C., Marie A., Acquaviva R., Cappello A.R., Deguin B. (2023). Iridoid- and Flavonoid-Enriched Fractions of *Cornus sanguinea* and *Cornus mas* Exert Antioxidant and Anti-Inflammatory Effects and Inhibit Key Enzymes in the Treatment of Metabolic Disorders. Food Funct..

[8] Shih P.-H., Yeh C.-T., Yen G.-C. (2007). Anthocyanins Induce the Activation of Phase II Enzymes through the Antioxidant Response Element Pathway against Oxidative Stress-Induced Apoptosis. J. Agric. Food Chem..

[9] Calfío C., Donoso F., Huidobro-Toro J.P. (2021). Anthocyanins Activate Membrane Estrogen Receptors with Nanomolar Potencies to Elicit a Nongenomic Vascular Response Via NO Production. JAHA.

[10] Lila M.A. (2004). Anthocyanins and Human Health: An In Vitro Investigative Approach. J. Biomed. Biotechnol..

[11] Wallace T.C. (2011). Anthocyanins in Cardiovascular Disease. Adv. Nutr..

[12] Lachowicz S., Bieniek A., Gil Z., Bielska N., Markuszewski B. (2019). Phytochemical Parameters and Antioxidant Activity of New Cherry Silverberry Biotypes (*Elaeagnus multiflora* Thunb.). Eur. Food Res. Technol..

[13] Szpadzik E., Krupa T., Molska-Kawulok K., Przybyłko S. (2022). Fruit Quality and Contents of Some Bioactive Compounds in Selected Czech Sweet Cherry (*Prunus avium* L.) Cultivars under Conditions of Central Poland. Agriculture.

[14] Unal N., Okatan V., Bilgin J., Kahramanoğlu I., Hajizadeh H.S. (2023). Impacts of Different Planting Times on Fruit Quality and Some Bioactive Contents of Different Strawberry Cultivars. Folia Hortic..

[15] Krupa T., Tomala K. (2021). Effect of Oxygen and Carbon Dioxide Concentration on the Quality of Minikiwi Fruits after Storage. Agronomy.

[16] Demasi S., Caser M., Scariot V. (2023). Hot and Cold Drying of Edible Flowers Affect Metabolite Patterns of Extracts and Decoctions. Folia Hortic..

[17] Tenuta M.C., Deguin B., Loizzo M.R., Cuyamendous C., Bonesi M., Sicari V., Trabalzini L., Mitaine-Offer A.-C., Xiao J., Tundis R. (2022). An Overview of Traditional Uses, Phytochemical Compositions and Biological Activities of Edible Fruits of European and Asian Cornus Species. Foods.

[18] Jaćimović V., Božović D., Ercisli S., Bosančić B., Necas T. (2020). Sustainable Cornelian Cherry Production in Montenegro: Importance of Local Genetic Resources. Sustainability.

[19] Szot I., Łysiak G.P., Sosnowska B. (2023). The Beneficial Effects of Anthocyanins from Cornelian Cherry (*Cornus mas* L.) Fruits and Their Possible Uses: A Review. Agriculture.

[20] Szot I., Łysiak G.P. (2022). Effect of the Climatic Conditions in Central Europe on the Growth and Yield of Cornelian Cherry Cultivars. Agriculture.

[21] Horbowicz M., Kosson R., Grzesiuk A., Dębski H. (2008). Anthocyanins of Fruits and Vegetables—Their Occurrence, Analysis and Role in Human Nutrition. J. Fruit. Ornam. Plant Res..

[22] Klymenko S. (2021). Phenological Stages of Development of *Cornus* L. S. Str. Species (*Cornaceae*) According to BBCH Scale. Agrobiodivers Improv. Nutr. Health Life Qual..

[23] Popović B.M., Štajner D., Slavko K., Sandra B. (2012). Antioxidant Capacity of Cornelian Cherry (*Cornus mas* L.)—Comparison between Permanganate Reducing Antioxidant Capacity and Other Antioxidant Methods. Food Chem..

[24] Łysiak G. (2022). Ornamental Flowers Grown in Human Surroundings as a Source of Anthocyanins with High Anti-Inflammatory Properties. Foods.

[25] Dzydzan O., Bila I., Kucharska A.Z., Brodyak I., Sybirna N. (2019). Antidiabetic Effects of Extracts of Red and Yellow Fruits of Cornelian Cherries (*Cornus mas* L.) on Rats with Streptozotocin-Induced Diabetes Mellitus. Food Funct..

[26] Chaves-Silva S., Santos A.L.D., Chalfun-Júnior A., Zhao J., Peres L.E.P., Benedito V.A. (2018). Understanding the Genetic Regulation of Anthocyanin Biosynthesis in Plants—Tools for Breeding Purple Varieties of Fruits and Vegetables. Phytochemistry.

[27] Borroto Fernández E.G., Mokhber A., Zeiser M., Laimer M. (2022). Phenotypic Characterization of a Wild-Type Population of Cornelian Cherries (*Cornus mas* L.) from Austria. Erwerbs-Obstbau.

[28] Martinović A., Cavoski I. (2020). The Exploitation of Cornelian Cherry (*Cornus mas* L.) Cultivars and Genotypes from Montenegro as a Source of Natural Bioactive Compounds. Food Chem..

[29] Milenkovic-Andjelkovic A., Radovanovic B., Andjelkovic M., Radovanovic A., Nikolic V., Randjelovic V. (2015). The Anthocyanin Content and Bioactivity of Cornelian Cherry (*Cornus mas*) and Wild Blackberry (*Rubus fruticosus*): Fruit Extracts from the Vlasina Region. Adv. Tech..

[30] Szczepaniak O.M., Kobus-Cisowska J., Kusek W., Przeor M. (2019). Functional Properties of Cornelian Cherry (*Cornus mas* L.): A Comprehensive Review. Eur. Food Res. Technol..

[31] Antolak H., Czyzowska A., Sakač M., Mišan A., Đuragić O., Kregiel D. (2017). Phenolic Compounds Contained in Little-Known Wild Fruits as Antiadhesive Agents Against the Beverage-Spoiling Bacteria *Asaia* spp. Molecules.

[32] Ochmian I., Oszmiański J., Lachowicz S., Krupa-Małkiewicz M. (2019). Rootstock Effect on Physico-Chemical Properties and Content of Bioactive Compounds of Four Cultivars Cornelian Cherry Fruits. Sci. Hortic..

[33] Sengul M., Eser Z., Ercisli S. (2014). Chemical Properties and Antioxidant Capacity of Cornelian Cherry Genotypes Grown in Coruh Valley of Turkey. ASPHC.

[34] Begic-Akagic A., Drkenda P., Vranac A., Orazem P., Hudina M. (2013). Influence of Growing Region and Storage Time on Phenolic Profile of Cornelian Cherry Jam and Fruit. Eur. J. Hortic. Sci..

[35] Manach C., Williamson G., Morand C., Scalbert A., Rémésy C. (2005). Bioavailability and Bioefficacy of Polyphenols in Humans. I. Review of 97 Bioavailability Studies. Am. J. Clin. Nutr..

[36] Oliveira H., Roma-Rodrigues C., Santos A., Veigas B., Brás N., Faria A., Calhau C., De Freitas V., Baptista P.V., Mateus N. (2019). GLUT1 and GLUT3 Involvement in Anthocyanin Gastric Transport- Nanobased Targeted Approach. Sci. Rep..

[37] Passamonti S., Vrhovsek U., Mattivi F. (2002). The Interaction of Anthocyanins with Bilitranslocase. Biochem. Biophys. Res. Commun..

[38] Talavéra S., Felgines C., Texier O., Besson C., Manach C., Lamaison J.-L., Rémésy C. (2004). Anthocyanins Are Efficiently Absorbed from the Small Intestine in Rats. J. Nutr..

[39] Mallery S.R., Budendorf D.E., Larsen M.P., Pei P., Tong M., Holpuch A.S., Larsen P.E., Stoner G.D., Fields H.W., Chan K.K. (2011). Effects of Human Oral Mucosal Tissue, Saliva and Oral Microflora on Intraoral Metabolism and Bioactivation of Black Raspberry Anthocyanins. Cancer Prev. Res..

[40] Han F., Yang P., Wang H., Fernandes I., Mateus N., Liu Y. (2019). Digestion and Absorption of Red Grape and Wine Anthocyanins through the Gastrointestinal Tract. Trends Food Sci. Technol..

[41] Keppler K., Humpf H.-U. (2005). Metabolism of Anthocyanins and Their Phenolic Degradation Products by the Intestinal Microflora. Bioorganic Med. Chem..

[42] David L., Danciu V., Moldovan B., Filip A. (2019). Effects of In Vitro Gastrointestinal Digestion on the Antioxidant Capacity and Anthocyanin Content of Cornelian Cherry Fruit Extract. Antioxidants.

[43] Seeram N.P., Schutzki R., Chandra A., Nair M.G. (2002). Characterization, Quantification, and Bioactivities of Anthocyanins in *Cornus* Species. J. Agric. Food Chem..

[44] Yousefi B., Abasi M., Abbasi M.M., Jahanban-Esfahlan R. (2015). Anti-Proliferative Properties of *Cornus mass* Fruit in Different Human Cancer Cells. Asian Pac. J. Cancer Prev..

[45] Odžaković B., Sailović P., Bodroža D., Kojić V., Jakimov D., Kukrić Z. (2021). Bioactive Components and Antioxidant, Antiproliferative, and Antihyperglycemic Activities of Wild Cornelian Cherry (*Cornus mas* L.). Maced. J. Chem. Chem. Eng..

[46] Jayaprakasam B., Vareed S.K., Olson L.K., Nair M.G. (2005). Insulin Secretion by Bioactive Anthocyanins and Anthocyanidins Present in Fruits. J. Agric. Food Chem..

[47] Świerczewska A., Buchholz T., Melzig M.F., Czerwińska M.E. (2019). In Vitro α-Amylase and Pancreatic Lipase Inhibitory Activity of *Cornus mas* L. and *Cornus alba* L. Fruit Extracts. J. Food Drug Anal..

[48] Zarei L., Sadrkhanlou R., Shahrooz R., Malekinejad H., Eilkhanizadeh B., Ahmadi A. (2014). Protective Effects of Vitamin E and *Cornus mas* Fruit Extract on Methotrexate-Induced Cytotoxicity in Sperms of Adult Mice. Vet. Res. Forum.

[49] Eshaghi M., Zare S., Banihabib N., Nejati V., Farokhi F., Mikaili P. (2012). Cardioprotective Effect of *Cornus mas* Fruit Extract against Carbon Tetrachloride Induced-Cardiotoxicity in Albino Rats. J. Basic Appl. Sci. Res..

[50] Moayed Alavian S., Banihabib N., Es. Haghi M., Panahi F. (2014). Protective Effect of *Cornus mas* Fruits Extract on Serum Biomarkers in CCl4-Induced Hepatotoxicity in Male Rats. Hepat. Mon..

[51] Somi M.H., Banihabib N., Dehghan G., Es. Haghi M., Panahi F. (2014). Hepatoprotective Effect of *Cornus mas* Fruits Extract Against Carbon Tetrachloride-Induced Hepatic Damage in Male Albino Rats. Thrita.

[52] Saei H., Hatami H., Azarmi M., Dehghan G. (2016). Hepatoprotective Effect of *Cornus mas* Fruit Extract on Serum Biomarkers in Methotrexate-Induces Liver Injury in Male Rats. Pharmacol. Line.

[53] Es Hagi M., Dehghan G., Banihabib N., Zare S., Mikalili P., Panahi F. (2014). Protective Effects of *Cornus mas* Fruit Extract on Carbon Tetrachloride Induced Nephrotoxicity in Rats. Indian. J. Nephrol..

[54] Francik R., Kryczyk J., Krośniak M., Berköz M., Sanocka I., Francik S. (2014). The Neuroprotective Effect of *Cornus mas* on Brain Tissue of Wistar Rats. Sci. World J..

[55] Darbandi N., Hashemi A., Noori M., Momeni H.R. (2016). Effect of *Cornus mas* Fruit Flavonoids on Memory Retention, Level of Plasma Glucose and Lipids in an Intracerebroventricular Streptozotocin-Induced Experimental Alzheimer’s Disease Model in Wistar Rats. EEB.

[56] Omelka R., Blahova J., Kovacova V., Babikova M., Mondockova V., Kalafova A., Capcarova M., Martiniakova M. (2020). Cornelian Cherry Pulp Has Beneficial Impact on Dyslipidemia and Reduced Bone Quality in Zucker Diabetic Fatty Rats. Animals.

[57] Nowak B., Matuszewska A., Szeląg A., Danielewski M., Dziewiszek W., Nikodem A., Filipiak J., Jędrzejuk D., Bolanowski M., Kucharska A.Z. (2022). *Cornelian cherry* (*Cornus mas* L.) Extract Reduces Cardiovascular Risk and Prevents Bone Loss in Ovariectomized Wistar Rats. J. Funct. Foods.

[58] Asgary S., Rafieian-Kopaei M., Shamsi F., Najafi S., Sahebkar A. (2014). Biochemical and Histopathological Study of the Anti-Hyperglycemic and Anti-Hyperlipidemic Effects of *Cornelian cherry* (*Cornus mas* L.) in Alloxan-Induced Diabetic Rats. J. Complement. Integr. Med..

[59] Capcarova M., Kalafova A., Schwarzova M., Schneidgenova M., Svik K., Prnova M.S., Slovak L., Kovacik A., Lory V., Zorad S. (2019). Cornelian Cherry Fruit Improves Glycaemia and Manifestations of Diabetes in Obese Zucker Diabetic Fatty Rats. Res. Vet. Sci..

[60] Jayaprakasam B., Olson L.K., Schutzki R.E., Tai M.-H., Nair M.G. (2006). Amelioration of Obesity and Glucose Intolerance in High-Fat-Fed C57BL/6 Mice by Anthocyanins and Ursolic Acid in *Cornelian cherry* (*Cornus mas*). J. Agric. Food Chem..

[61] Sozański T., Kucharska A.Z., Szumny A., Magdalan J., Bielska K., Merwid-Ląd A., Woźniak A., Dzimira S., Piórecki N., Trocha M. (2014). The Protective Effect of the *Cornus mas* Fruits (*Cornelian cherry*) on Hypertriglyceridemia and Atherosclerosis through PPARα Activation in Hypercholesterolemic Rabbits. Phytomedicine.

[62] Sozański T., Kucharska A., Dzimira S., Magdalan J., Szumny D., Matuszewska A., Nowak B., Piórecki N., Szeląg A., Trocha M. (2018). Loganic Acid and Anthocyanins from *Cornelian cherry* (*Cornus mas* L.) Fruits Modulate Diet-Induced Atherosclerosis and Redox Status in Rabbits. Adv. Clin. Exp. Med..

[63] Sozański T., Kucharska A.Z., Rapak A., Szumny D., Trocha M., Merwid-Ląd A., Dzimira S., Piasecki T., Piórecki N., Magdalan J. (2016). Iridoid–Loganic Acid versus Anthocyanins from the *Cornus mas* Fruits (*Cornelian cherry*): Common and Different Effects on Diet-Induced Atherosclerosis, PPARs Expression and Inflammation. Atherosclerosis.

[64] Danielewski M., Kucharska A.Z., Matuszewska A., Rapak A., Gomułkiewicz A., Dzimira S., Dzięgiel P., Nowak B., Trocha M., Magdalan J. (2021). *Cornelian cherry* (*Cornus mas* L.) Iridoid and Anthocyanin Extract Enhances PPAR-α, PPAR-γ Expression and Reduces I/M Ratio in Aorta, Increases LXR-α Expression and Alters Adipokines and Triglycerides Levels in Cholesterol-Rich Diet Rabbit Model. Nutrients.

[65] Asgary S., Rafieian-Kopaei M., Adelnia A., Kazemi S., Shamsi F. (2010). Comparing the Effects of Lovastatin and *Cornus mas* Fruit on Fibrinogen Level in Hypercholesterolemic Rabbits. ARYA Atheroscler..

[66] Abdollahi B., Mesgari Abbasi M., Zakeri Milani P., Sadat Nourdadgar A., Banan Khojasteh S.M., Nejati V. (2014). Hydro-Methanolic Extract of *Cornus mas* L. and Blood Glucose, Lipid Profile and Hematological Parameters of Male Rats. Iran. Red. Crescent Med. J..

[67] Szumny D., Sozański T., Kucharska A.Z., Dziewiszek W., Piórecki N., Magdalan J., Chlebda-Sieragowska E., Kupczynski R., Szeląg A., Szumny A. (2015). Application of Cornelian Cherry Iridoid-Polyphenolic Fraction and Loganic Acid to Reduce Intraocular Pressure. Evid.-Based Complement. Altern. Med..

[68] Sangsefidi Z.S., Hosseinzadeh M., Ranjbar A.M., Akhondi-Meybodi M., Fallahzadeh H., Mozaffari-Khosravi H. (2019). The Effect of Total Anthocyanin-Base Standardized (*Cornus mas* L.) Fruit Extract on Liver Function, Tumor Necrosis Factor α, Malondealdehyde, and Adiponectin in Patients with Non-Alcoholic Fatty Liver: A Study Protocol for a Double-Blind Randomized Clinical Trial. Nutr. J..

[69] Sangsefidi Z.S., Yarhosseini F., Hosseinzadeh M., Ranjbar A., Akhondi-Meybodi M., Fallahzadeh H., Mozaffari-Khosravi H. (2021). The Effect of (*Cornus mas* L.) Fruit Extract on Liver Function among Patients with Nonalcoholic Fatty Liver: A Double-blind Randomized Clinical Trial. Phytother. Res..

[70] Soltani R., Gorji A., Asgary S., Sarrafzadegan N., Siavash M. (2015). Evaluation of the Effects of *Cornus mas* L. Fruit Extract on Glycemic Control and Insulin Level in Type 2 Diabetic Adult Patients: A Randomized Double-Blind Placebo-Controlled Clinical Trial. Evid.-Based Complement. Altern. Med..

[71] Asgary S., Kelishadi R., Rafieian-Kopaei M., Najafi S., Najafi M., Sahebkar A. (2013). Investigation of the Lipid-Modifying and Antiinflammatory Effects of *Cornus mas* L. Supplementation on Dyslipidemic Children and Adolescents. Pediatr. Cardiol..

[72] Yarhosseini F., Sangouni A.A., Sangsefidi Z.S., Hosseinzadeh M., Akhondi-Meybodi M., Ranjbar A., Fallahzadeh H., Mozaffari-Khosravi H. (2023). Effect of *Cornus mas* L. Fruit Extract on Blood Pressure, Anthropometric and Body Composition Indices in Patients with Non-Alcoholic Fatty Liver Disease: A Double-Blind Randomized Controlled Trial. Clin. Nutr. ESPEN.

[73] Dadkhah N., Shirani M., Etemadifar S., Mirtalebi M. (2017). The Effect of *Cornus mas* in Preventing Recurrent Urinary Tract Infections in Women. Future Nat. Prod..

[74] Seeram N. (2001). Cyclooxygenase Inhibitory and Antioxidant Cyanidin Glycosides in Cherries and Berries. Phytomedicine.

[75] Wang H., Nair M.G., Strasburg G.M., Chang Y.-C., Booren A.M., Gray J.I., DeWitt D.L. (1999). Antioxidant and Antiinflammatory Activities of Anthocyanins and Their Aglycon, Cyanidin, from Tart Cherries. J. Nat. Prod..

[76] Szaniawska M., Taraba A., Szymczyk K. (2015). Structure, Properties and Application of Anthocyanins. Eng. Sci. Technol..

[77] Sarma A.D., Sharma R. (1999). Anthocyanin-DNA Copigmentation Complex: Mutual Protection against Oxidative Damage. Phytochemistry.

[78] Szczepaniak O., Ligaj M., Stuper-Szablewska K., Kobus-Cisowska J. (2022). Genoprotective Effect of Cornelian Cherry (*Cornus mas* L.) Phytochemicals, Electrochemical and Ab Initio Interaction Study. Biomed. Pharmacother..

[79] Nomi Y., Iwasaki-Kurashige K., Matsumoto H. (2019). Therapeutic Effects of Anthocyanins for Vision and Eye Health. Molecules.

[80] Renis M., Calandra L., Scifo C., Tomasello B., Cardile V., Vanella L., Bei R., Fauci L.L., Galvano F. (2008). Response of Cell Cycle/Stress-Related Protein Expression and DNA Damage upon Treatment of CaCo2 Cells with Anthocyanins. Br. J. Nutr..

[81] Wang L.-S., Stoner G.D. (2008). Anthocyanins and Their Role in Cancer Prevention. Cancer Lett..

[82] Łysiak G.P., Szot I. (2023). The Possibility of Using Fruit-Bearing Plants of Temperate Climate in the Treatment and Prevention of Diabetes. Life.

[83] Tian Y., Du H., Wang L., Li S., Zhang L., Zhang L. (2020). Nitrite Scavenging and Inhibition of *N* -Nitrosamines Formation by Phenolic Extracts From *Diospyros Lotus* L. Leaves and Active Ingredients. Nat. Prod. Commun..

[84] Rafieian-Kopaei M., Asgary S., Adelnia A., Setorki M., Khazaei M., Somayek K., Shamsi F. (2011). The Effects of Cornelian Cherry on Atherosclerosis and Atherogenic Factors in Hypercholesterolemic Rabbits. J. Med. Plants Res..

[85] Liu C., Sun J., Lu Y., Bo Y. (2016). Effects of Anthocyanin on Serum Lipids in Dyslipidemia Patients: A Systematic Review and Meta-Analysis. PLoS ONE.

[86] Krzyściak P., Krośniak M., Gąstoł M., Ochońska D., Krzyściak W. (2011). Antimicrobial Activity of Cornelian Cherry (*Cornus mas* L.). Postępy Fitoter..

[87] West B.J., Deng S., Jensen C.J., Palu A.K., Berrio L.F. (2012). Antioxidant, Toxicity, and Iridoid Tests of Processed Cornelian Cherry Fruits: Tests of Processed Cornelian Cherry Fruits. Int. J. Food Sci. Technol..

[88] European Food Safety Authority Scientific Opinion on the Re-Evaluation of Anthocyanins (E 163) as a Food Additive. https://efsa.onlinelibrary.wiley.com/doi/epdf/10.2903/j.efsa.2013.3145.

[89] Srinivas N.R. (2013). Cranberry Juice Ingestion and Clinical Drug-Drug Interaction Potentials; Review of Case Studies and Perspectives. J. Pharm. Pharm. Sci..

